# Quantitative measurement of empathy and analysis of its correlation to clinical factors in korean patients with chronic diseases

**DOI:** 10.1186/s40885-023-00246-5

**Published:** 2023-07-15

**Authors:** Ran Heo, Jinho Shin, Byung Sik Kim, Hyun-Jin Kim, Kye-Yeung Park, Hoon-Ki Park, Yu Mi Kim, Seon Young Hwang, Stewart W. Mercer

**Affiliations:** 1 Division of Cardiology, Department of Internal Medicine, Hanyang University College of Medicine, Seoul, Republic of Korea; 2 Division of Cardiology, Department of Internal Medicine, Hanyang University Guri Hospital, Hanyang University College of Medicine, Guri, Republic of Korea; 3 Department of Family Medicine, Hanyang University College of Medicine, Seoul, Republic of Korea; 4 Department of Preventive Medicine, Hanyang University College of Medicine, Seoul, Republic of Korea; 5 School of Nursing, Hanyang University, Seoul, Republic of Korea; 6 Centre for Population Health Sciences, Usher Institute, University of Edinburgh College of Medicine and Veterinary Medicine, Edinburgh, Scotland

**Keywords:** Empathy, Chronic disease, Patient-centered care

## Abstract

**Background:**

Empathy is the core of the physician-patient relationship. The Consultation and Relational Empathy (CARE) measure is a useful tool for assessing patient-rated empathy. There have been scarce data on empathy in chronic disease patients in Korea. We aim to evaluate empathy using the Korean CARE measure in patients from various clinical environments and the factors influencing the degree of empathy in patients with chronic disease.

**Methods:**

Data were collected from patients with chronic diseases. Patients were from primary, secondary, and tertiary clinics. Characteristics of the patients, physicians, and disease status were collected. The difference in CARE score was studied according to the clinical factors.

**Results:**

A total of 162 patients with chronic diseases were included. About 60% of patients were male. The mean age was 62 years. They had an average number of 2.6 diseases. More than half of patients experienced overt cardiovascular disease. About half of them had a history of hospitalization due to cardiovascular disease. The overall average CARE score was 45.6 ± 7.0. The CARE score was not significantly different according to the characteristics of the patient, physician, or disease status. Regarding marital status, the CARE score was significantly lower for the small number of patients (n = 4, 2.5%) who refused to provide their marital status than for other groups. Except for four patients, there was no significant difference in the CARE score among married, unmarried, or divorced groups. This trend was maintained in hypertensive patients.

**Conclusions:**

The Korean CARE measure could assess patient-rated empathy in various clinical practices. The empathy of patients was high regardless of multiple factors.

**Supplementary Information:**

The online version contains supplementary material available at 10.1186/s40885-023-00246-5.

## Background

Cardiovascular disease is the second leading cause of death in Korea. The cardiovascular mortality rate has increased over 10 years from 44 to 60.2% from 2007 to 2017 [1]. In a narrow perspective of secondary prevention, cardiovascular disease refers to ischemic heart disease, valve disease, arrhythmia, and heart failure. It includes preceding chronic diseases, such as hypertension, diabetes, and dyslipidemia, in a broad sense of primary prevention. As the elderly population increases and the prevalence of antecedent diseases due to unhealthy lifestyles increases, the economic burden caused by cardiovascular diseases in Korea is expected to continue to increase.

Hypertension is one of the most important risk factors for cardiovascular disease. However, the treatment control rate of hypertension is less than 50% [2], facing serious challenges. Moreover, since the improvement in the hypertension control rate in Korea has been minimal over the past 10 years, more fundamental measures are needed. Improving such a low treatment rate is an urgent task.

In Korea, efforts are being made to implement the cardiovascular disease treatment guidelines properly in actual practice by introducing the concept of patient-centered medicine. In patient-centered medicine, it is crucial to motivate patients based on patient-centered clinical communication [3]. In secondary prevention patients whose quality of life was markedly reduced due to symptoms, frequent hospitalization, and risk of death, maintaining the motivation for the treatment is critical.

Among the various elements, empathy is the most initial and critical step in the physician-patient relationship. Without empathy, patients might even resist the provision of therapeutic information. Evaluation of empathy in medical interviews includes methods such as third-party evaluation of physicians or medical staff, self-questioning by medical staff, and patient questionnaires. The former options are widely used to measure various educational goals or effects related to empathy. However, empathy evaluation by the patient seems to be the most desirable starting point in patient-centered medicine. The Consultation and Relational Empathy (CARE) measure questionnaire was developed in the United Kingdom to measure patient perceptions of relational empathy and communication during a consultation with physicians [4]. It has been translated and validated in many languages [5–7]. Recently, our group validated the Korean version of the CARE measure [8].

We aimed to evaluate empathy using the Korean CARE measure in patients from various environments, including domestic primary, secondary, and tertiary medical institutions. Our second aim was to compare and evaluate the factors influencing the degree of empathy with cardiovascular disease patients.

## Methods

### Ethical statements

The Institutional Review Board of Hanyang University Hospital approved this study (No. 2020–09‑007). The study was conducted in compliance with the Helsinki Declaration. Informed consent was submitted by all subjects when they were enrolled.

### Study subjects

Enrolled patients were 20 years or older and visited one of three clinics. Those were a tertiary and a secondary cardiology outpatient clinic, or a family medicine outpatient clinic at the tertiary care hospital, from February to July 2021.

The main diagnosis of patients was angina, myocardial infarction, valvular heart disease, heart failure, arrhythmia, hypertension, or hyperlipidemia. Those who could read and communicate in Korean and gave their written consent to participate in the study were included. Those with a brain disease, a psychiatric disorder, or those who refused to participate in the study were excluded.

### Data collection

A research nurse explained the purpose of the study in a one-on-one setting to the study subjects. An anonymous paper and pencil version of the Korean CARE measure was completed by the patients after their clinical encounter. A numeric score for empathy was calculated based on responses to the CARE measure. Characteristics of the patients, physicians, and disease status were collected. For the patients, demographic information such as age and sex, and social information including employment, marital, and educational status were collected. For the physicians, the duration of their medical carrier, sex, and characteristics of their specialty were collected. Regarding the disease status, medical history for hypertension, dyslipidemia, diabetes mellitus, valvular heart disease, arrhythmia, angina, myocardial infarction, heart failure, cerebral stroke, and history of admission were collected.

### Data analysis

The 10 questions in the CARE measure are rated by patients in response to the questions with a score of 1 for “poor” and 5 for “excellent.” The total score is then calculated by adding up the 10-item scores (range, 10–50) (Supplements 1, 2).

Student t-test and chi-square test were used to assess potential differences between the two groups. Analysis of variance analysis was used for comparing continuous variables among multiple groups. Analyses were performed using IBM SPSS ver. 27.0 (IBM Corp).

## Results

A total of 162 patients with chronic diseases were included. About 60% of patients were male. Their mean age was 62.2 ± 13.9 years. Most were married, nonemployed, and had a diverse education status. They had an average number of 2.6 diseases. More than half of patients experienced overt cardiovascular diseases such as heart failure, myocardial infarction, angina, and cerebral stroke. About half of them had a history of hospitalization due to cardiovascular disease. The characteristics of patients are shown in Table 1.

**Table 1:** Patient demographic characteristics and empathy scores according to the Korean version of the CARE measure

Characteristic	No (%)	CARE score (mean ± SD)	P-value
Age (yr)			0.181
≤ 39	11 (6.8)	41.8 ± 11.4	
40–64	83 (51.2)	45.8 ± 6.2	
≥ 65	68 (42.0)	46.0 ± 7.1	
Sex			0.853
Male	96 (59.3)	45.8 ± 6.9	
Female	66 (40.7)	45.4 ± 7.3	
Department visited			0.426
Cardiovascular (tertiary)	112 (69.1)	46.0 ± 6.3	
Cardiovascular (secondary)	30 (18.5)	44.1 ± 7.1	
Family medicine (primary)	20 (12.4)	45.6 ± 10.3	
Marital status			0.002
Married	136 (84.0)	45.9 ± 6.8	
Divorced	10 (6.2)	47.4 ± 4.0	
Unmarried	12 (7.4)	45.2 ± 4.7	
Not available	4 (2.4)	32.8 ± 15.2	
Education			0.723
Elementary school or below	39 (24.1)	46.6 ± 5.8	
Middle and high school	53 (32.7)	45.2 ± 7.8	
College or above	32 (19.7)	45.8 ± 4.9	
Not available	38 (23.5)	45.0 ± 8.7	
Occupation			0.608
Yes	61 (37.7)	44.9 ± 7.6	
No	95 (58.6)	46.1 ± 6.8	
Not available	6 (3.7)	45.2 ± 5.3	
Treatment setting			0.609
Primary prevention	74 (45.7)	45.7 ± 7.4	
Secondary prevention	88 (54.3)	45.5 ± 6.8	
No. of disease			0.132
1 or 2	77 (47.5)	45.1 ± 7.7	
≥ 3	85 (52.5)	46.1 ± 6.4	
History of admission for cardiovascular disease			0.099
Yes	83 (51.2)	46.0 ± 6.4	
No	79 (48.8)	45.2 ± 7.7	
Total	162 (100)	45.6 ± 7.0	-

CARE, Consultation and Relational Empathy; SD, standard deviation

The overall average CARE score was 45.6 ± 7.0. The CARE score was not different according to the patient’s age, sex, social factors, type of clinic they visited (primary vs. secondary vs. tertiary, 45.6 vs. 44.1 vs. 46.0; P = 0.426), treatment setting (primary vs. secondary prevention, 45.7 vs. 45.5; P = 0.609) or the presence of a history of admission for cardiovascular disease (46.0 vs. 45.2, P = 0.099), and the number of diseases (1 or 2 vs. 3 or more, 45.1 vs. 46.1; P = 0.132). Marital status demonstrated a significant CARE score difference. The score was lower for the patients who refused to provide their marital status than for other groups, although the number of that group was small (n = 4) (Table 1). Besides those 4 subjects, there was no significant difference in the CARE score among married, unmarried, or divorced groups (P = 0.713). We compared the CARE score according to the characteristics of physicians. The CARE score was not affected by those factors either (Table 2).

**Table 2:** Physician characteristics and empathy scores according to a Korean version of the CARE measure (n = 162)

Characteristic	No (%)	CARE score (mean ± SD)	P-value
Experience (yr)			0.674
≤ 14	92 (56.8)	45.5 ± 6.4	
> 14	70 (43.2)	45.7 ± 7.8	
Sex			0.590
Male	131 (80.9)	45.6 ± 7.2	
Female	31 (19.1)	46.6 ± 6.6	
Specialty			0.153
Intervention	60 (37.0)	44.7 ± 7.8	
Nonintervention	102 (63.0)	46.2 ± 6.5	

CARE, Consultation and Relational Empathy; SD, standard deviation

Hypertension is the most frequent disease in our study (n = 143, 88.3%). We assessed the CARE score in this subgroup. Among hypertensive patients, there was also no significant factor for the CARE score difference between groups according to the patient’s or the physician’s characteristics, except marital status (Tables 3 and 4). Like the CARE score of the overall group, the score was lower for the patients who refused to provide their marital status than other groups, however other than them there was no difference in the CARE score regarding marital status (P = 0.915). The CARE score was not different between patients receiving treatment for primary prevention and secondary prevention (45.3 vs. 45.5, P = 0.504) nor between those with a history of hospitalization for cardiovascular disease and those without (46.0 vs. 44.8, P = 0.104). When analyzing the difference according to the characteristics of the physician, there was no difference according to the physician’s sex, specialty, or duration of the experience (Table 4).

**Table 3:** Patient demographic characteristics and empathy scores according to a Korean version of the CARE measure in hypertensive patients

Characteristic	No (%)	CARE score (mean ± SD)	P-value
Age (yr)			0.171
≤ 39	9 (6.3)	41.1 ± 12.4	
40–64	72 (50.3)	45.7 ± 6.0	
≥ 65	62 (43.4)	45.8 ± 7.3	
Sex			0.964
Male	88 (61.5)	45.6 ± 7.1	
Female	55 (38.5)	45.2 ± 7.3	
Department visited			0.466
Cardiovascular (tertiary)	104 (72.7)	45.9 ± 6.5	
Cardiovascular (secondary)	30 (21.0)	44.1 ± 7.1	
Family medicine (primary)	9 (6.3)	44.8 ± 13.2	
Marital status			0.004
Married	120 (83.9)	45.8 ± 6.8	
Divorced	8 (5.6)	46.8 ± 4.2	
Unmarried	11 (7.7)	45.6 ± 4.7	
Not available	4 (2.8)	32.8 ± 15.2	
Education			0.766
Elementary school or below	36 (25.2)	46.4 ± 5.9	
Middle and high school	49 (34.2)	45.4 ± 7.5	
College or above	23 (16.1)	45.4 ± 5.0	
Not available	35 (24.5)	44.6 ± 8.9	
Occupation			0.424
Yes	53 (37.1)	45.0 ± 7.5	
No	84 (58.7)	45.7 ± 7.1	
Not available	6 (4.2)	45.2 ± 5.3	
Treatment setting			0.504
Primary prevention	59 (41.3)	45.3 ± 7.5	
Secondary prevention	84 (58.7)	45.5 ± 6.9	
No. of disease			0.100
1 or 2	60 (42.0)	44.6 ± 8.0	
≥ 3	83 (58.0)	46.1 ± 6.4	
History of admission for cardiovascular disease			0.104
Yes	77 (53.8)	46.0 ± 6.5	
No	66 (46.2)	44.8 ± 7.8	
Total	143	45.4 ± 7.1	-

CARE, Consultation and Relational Empathy; SD, standard deviation

**Table 4:** Physician characteristics and empathy scores according to a Korean version of the CARE measure in hypertensive patients (n = 143)

Characteristic	No (%)	CARE score (mean ± SD)	P-value
Experience (yr)			0.782
≤ 14	84 (58.7)	45.4 ± 6.6	
> 14	59 (41.3)	45.6 ± 7.9	
Sex			0.550
Male	112 (78.3)	45.4 ± 7.3	
Female	31 (21.7)	45.6 ± 6.6	
Specialty			0.179
Intervention	60 (42.0)	44.7 ± 7.8	
Nonintervention	83 (58.0)	46.0 ± 6.6	

CARE, Consultation and Relational Empathy; SD, standard deviation

## Discussion

In this study, we evaluated patients’ perception of empathy from physicians with the Korean CARE measure for the first time. Overall CARE score was high regardless of the type of clinic, treatment setting (primary vs. secondary prevention), or other factors of patient or physicians.

Empathy is the ability to understand the psychological state or to respond emotional suffering of others [9]. Empathy is crucial to the development of the therapeutic relationship. Empathy in medical care is associated with many benefits including improved patient satisfaction, fewer medical errors, and positive effect on treatment outcomes [10–12]. Empathy is especially valuable in patients with chronic diseases to improve their adherence to the treatment. However, despite its importance, only a few studies were done in a clinical setting. Kim and Park [13] studied 150 rehabilitation patients and found that affective empathy, not cognitive empathy, was related to patient compliance. Choi et al. [14] studied 267 patients who underwent bronchoscopy. Verbal empathy and touch given by a physician before an exam reduced anxiety in patients with high baseline anxiety levels. Empathy was assessed by the patients in both studies. However, methods might be too simple like a visual analog scale [14], or complicated to use in clinics like 26-item questionnaires [13]. In cardiovascular disease, there is still scarce data.

There have been tools to measure empathy [15, 16]. However, there are concerns that the items included in these scales are too complicated to use in clinical practice or have generally been determined by professionals and may therefore fail to reflect the perspective of patients [17]. The CARE measure is simple to use and evaluated by patients, therefore is a helpful tool for patient-centered medicine.

The CARE measure in other languages has been used in chronic disease patients [5, 18, 19]. The CARE score was higher when patients were older or follow-up patients. The social factors including education or marital status were not affecting the CARE score. The average CARE scores of those studies were lower than in this study. Some studies demonstrated that income was not related to the CARE score. Some studies showed that longer consultations with multiple problems are related to higher CARE scores. One meta-analysis also demonstrated that longer consultations and female practitioners were related to the higher CARE score [20].

Recently the Korean version of the CARE has been developed and validated [8]. We aimed to search for factors related to the patient’s perception of empathy, so we could use it to construct a better patient-physician relationship which is critical in chronic disease. In this study, the overall CARE score was higher than in the previous studies. It might be related to the older population of this study, as age was related to the higher CARE score [5, 19]. Also, our patients were mostly follow-up patients, which was a factor for the higher CARE score compared to the new patient [5, 19]. However, the other study showed lower CARE scores in patients with more than 6 months of follow-up [18]. Regarding socioeconomic status (SES), some studies showed that it was not related to the CARE score [5]. However, one meta-analysis found that the patients with low SES experienced lower empathy from clinicians compared to patients whose SES was not low [21].

The difference in this study was that marital status was related to the CARE score. Patients who refused to mention their marital status showed lower CARE scores. However, except for a small number of those patients, marital status did not affect the CARE score significantly in overall and hypertensive patients. Generally, married patients demonstrated better clinical outcomes regardless of their cultural background [22–25]. One study conducted with Asian patients showed that being unmarried, as well as its unmarried subcategories, was positively associated with total and cause-specific mortality [24]. As our study involved patients who were older and with a higher prevalence of advanced cardiovascular disease, the impact of social factors might have worked differently than in previous studies.

Previous studies reported a lower cardiovascular event rate when the CARE score exceeded 46 [26]. Considering this, the CARE score of this study was high and could be related to the future favorable clinical outcome. In a population-based prospective cohort study of 628 diabetic patients, higher empathy scores were associated with a lower risk of cardiovascular events (although statistically insignificant) and a lower risk of all-cause mortality in 10-year follow-up [26]. Further studies are needed to investigate the potential role of the Korean CARE measure as a predictor for the clinical outcome.

Our study includes a diverse clinical environment, multiple chronic diseases, adequate representation of both sexes, and detailed delineation of advanced cardiovascular disease status. To our knowledge, this study is the first to demonstrate patient-rated empathy using the Korean CARE measure in chronic disease patients from various clinical practices. Our study, however, is not without limitations. One limitation is that the patients were mainly follow-up patients, which might not be reflected the characteristics of new patients. However, our study aimed to assess factors for the CARE score in chronic disease patients. Therefore, it might be hard to avoid. This study included a small number of subjects to represent the status of chronic cardiovascular disease in Korea. Therefore, the result of this study should be applied with caution. Further studies are needed with a larger number of patients. In future studies, the CARE measure could be used for first-time patients to compare the differences with this study. Some of the factors to the CARE score, such as income and the nature of consultation, were not available in our study. This might be included in future studies.

## Conclusions

We assessed the patient-rated empathy with the Korean CARE measure. The CARE score was relatively high regardless of the characteristics of patients or physicians, and disease status. This is the first milestone in measuring patient perception of a physician’s empathy in a Korean medical environment.

## Electronic supplementary material

Below is the link to the electronic supplementary material.

**Supplement 1.** CARE questionnaire in Korean

**Supplement 2.** CARE questionnaire in English

## Data Availability

The datasets are not publicly available but are available from the corresponding author upon reasonable request.

## Abbreviations

CARE: Consultation and Relational Empathy
SD: Standard deviation
SES: Socioeconomic status

## References

[1] Korea Disease Control and Prevention Agency. Chronic disease management research & development [Internet]. Korea Disease Control and Prevention Agency; [cited 2023 Jan 2]. Available from: https://www.kdca.go.kr/contents.es?mid=a30322000000.

[2] Kim HC, Lee H, Lee HH, Seo E, Kim E, Han J (2022). Korea hypertension fact sheet 2021: analysis of nationwide population-based data with special focus on hypertension in women. Clin Hypertens.

[3] Ihm SH, Kim KI, Lee KJ, Won JW, Na JO, Rha SW (2022). Interventions for adherence improvement in the primary prevention of cardiovascular diseases: expert consensus statement. Korean Circ J.

[4] Mercer SW, Maxwell M, Heaney D, Watt GC (2004). The consultation and relational empathy (CARE) measure: development and preliminary validation and reliability of an empathy-based consultation process measure. Fam Pract.

[5] Mercer SW, Fung CS, Chan FW, Wong FY, Wong SY, Murphy D (2011). The chinese-version of the CARE measure reliably differentiates between doctors in primary care: a cross-sectional study in Hong Kong. BMC Fam Pract.

[6] Aomatsu M, Abe H, Abe K, Yasui H, Suzuki T, Sato J (2014). Validity and reliability of the japanese version of the CARE measure in a general medicine outpatient setting. Fam Pract.

[7] van Dijk I, Scholten Meilink Lenferink N, Lucassen PL, Mercer SW, van Weel C, Olde Hartman TC (2017). Reliability and validity of the dutch version of the Consultation and Relational Empathy measure in primary care. Fam Pract.

[8] Park KY, Shin J, Park HK, Kim YM, Hwang SY, Shin JH (2022). Validity and reliability of a korean version of the Consultation and Relational Empathy (CARE) measure. BMC Med Educ.

[9] Coplan A. Understanding empathy: its features and effects. In: Coplan A, Goldie P, editors. Empathy: philosophical and psychological perspectives. Oxford University Press; 2011. pp. 2–18.

[10] Riess H (2017). The science of empathy. J Patient Exp.

[11] Kim SS, Kaplowitz S, Johnston MV (2004). The effects of physician empathy on patient satisfaction and compliance. Eval Health Prof.

[12] Hojat M, Louis DZ, Markham FW, Wender R, Rabinowitz C, Gonnella JS (2011). Physicians’ empathy and clinical outcomes for diabetic patients. Acad Med.

[13] Kim SS, Park BK (2008). Patient-perceived communication styles of physicians in rehabilitation: the effect on patient satisfaction and compliance in Korea. Am J Phys Med Rehabil.

[14] Choi SM, Lee J, Park YS, Lee CH, Lee SM, Yim JJ (2016). Effect of verbal empathy and touch on anxiety relief in patients undergoing flexible bronchoscopy: can empathy reduce patients’. anxiety? Respiration.

[15] La Monica EL (1981). Construct validity of an empathy instrument. Res Nurs Health.

[16] Hojat M, Gonnella JS, Nasca TJ, Mangione S, Vergare M, Magee M (2002). Physician empathy: definition, components, measurement, and relationship to gender and specialty. Am J Psychiatry.

[17] Reynolds W. The measurement and development of empathy in nursing. Routledge; 2000.

[18] Hanževački M, Jakovina T, Bajić Ž, Tomac A, Mercer S (2015). Reliability and validity of the croatian version of Consultation and Relational Empathy (CARE) measure in primary care setting. Croat Med J.

[19] Mercer SW, Murphy DJ (2008). Validity and reliability of the CARE measure in secondary care. Clin Gov Int J.

[20] Howick J, Steinkopf L, Ulyte A, Roberts N, Meissner K (2017). How empathic is your healthcare practitioner?: a systematic review and meta-analysis of patient surveys. BMC Med Educ.

[21] Roberts BW, Puri NK, Trzeciak CJ, Mazzarelli AJ, Trzeciak S (2021). Socioeconomic, racial and ethnic differences in patient experience of clinician empathy: results of a systematic review and meta-analysis. PLoS ONE.

[22] August KJ, Sorkin DH (2010). Marital status and gender differences in managing a chronic illness: the function of health-related social control. Soc Sci Med.

[23] Yim HJ, Park HA, Kang JH, Kim KW, Cho YG, Hur YI (2012). Marital status and health behavior in middle-aged korean adults. Korean J Fam Med.

[24] Leung CY, Huang HL, Abe SK, Saito E, Islam MR, Rahman MS (2022). Association of marital status with total and cause-specific mortality in Asia. JAMA Netw Open.

[25] Schultz WM, Hayek SS, Samman Tahhan A, Ko YA, Sandesara P, Awad M (2017). Marital status and outcomes in patients with cardiovascular disease. J Am Heart Assoc.

[26] Dambha-Miller H, Feldman AL, Kinmonth AL, Griffin SJ (2019). Association between primary care practitioner empathy and risk of cardiovascular events and all-cause mortality among patients with type 2 diabetes: a population-based prospective cohort study. Ann Fam Med.

